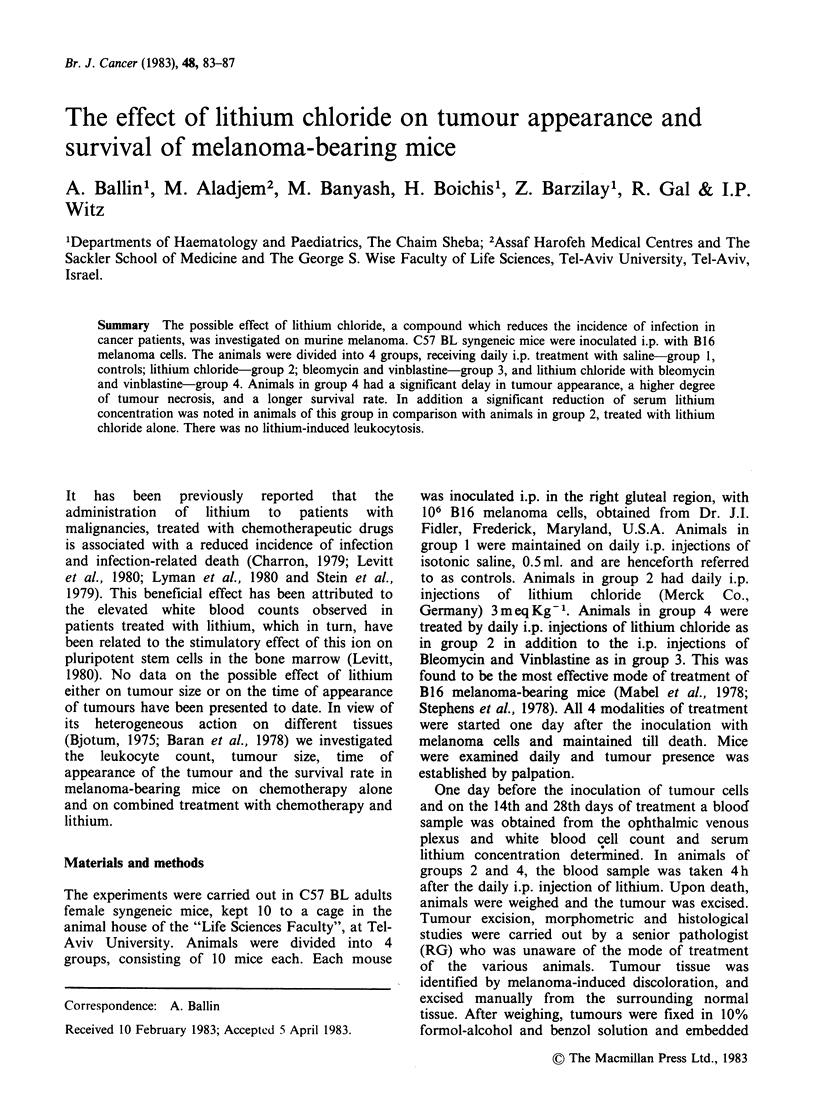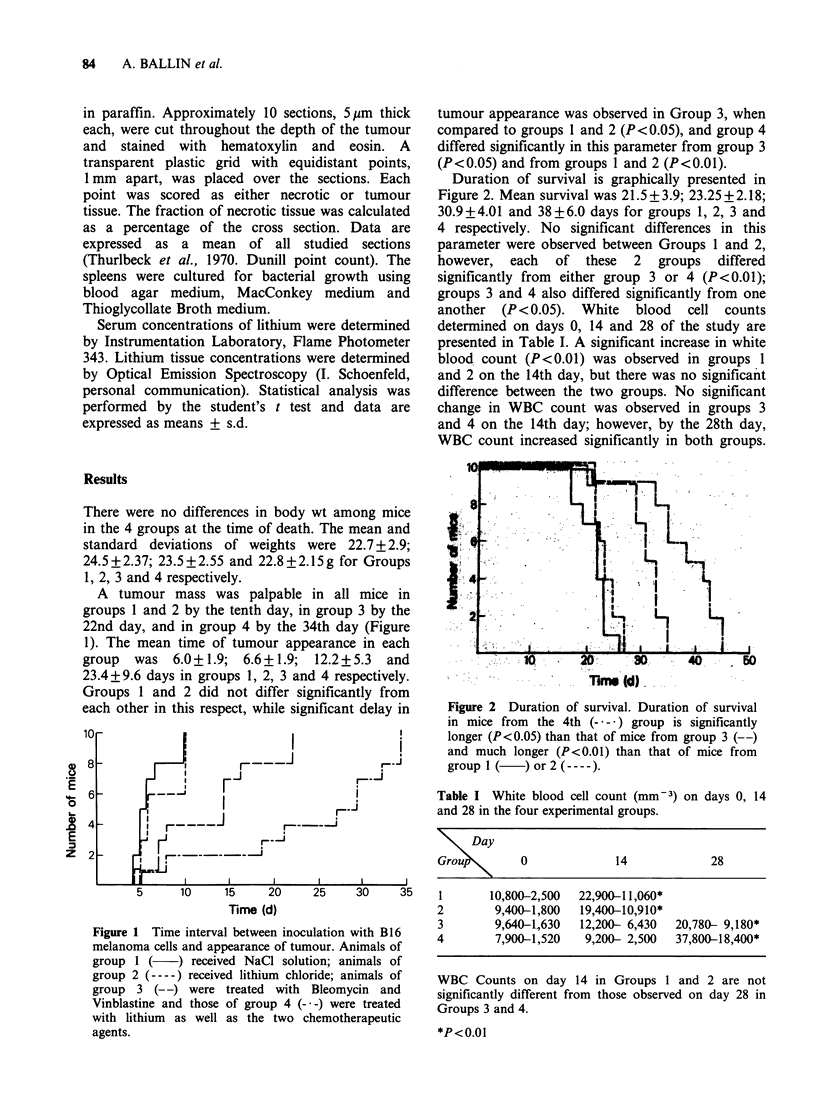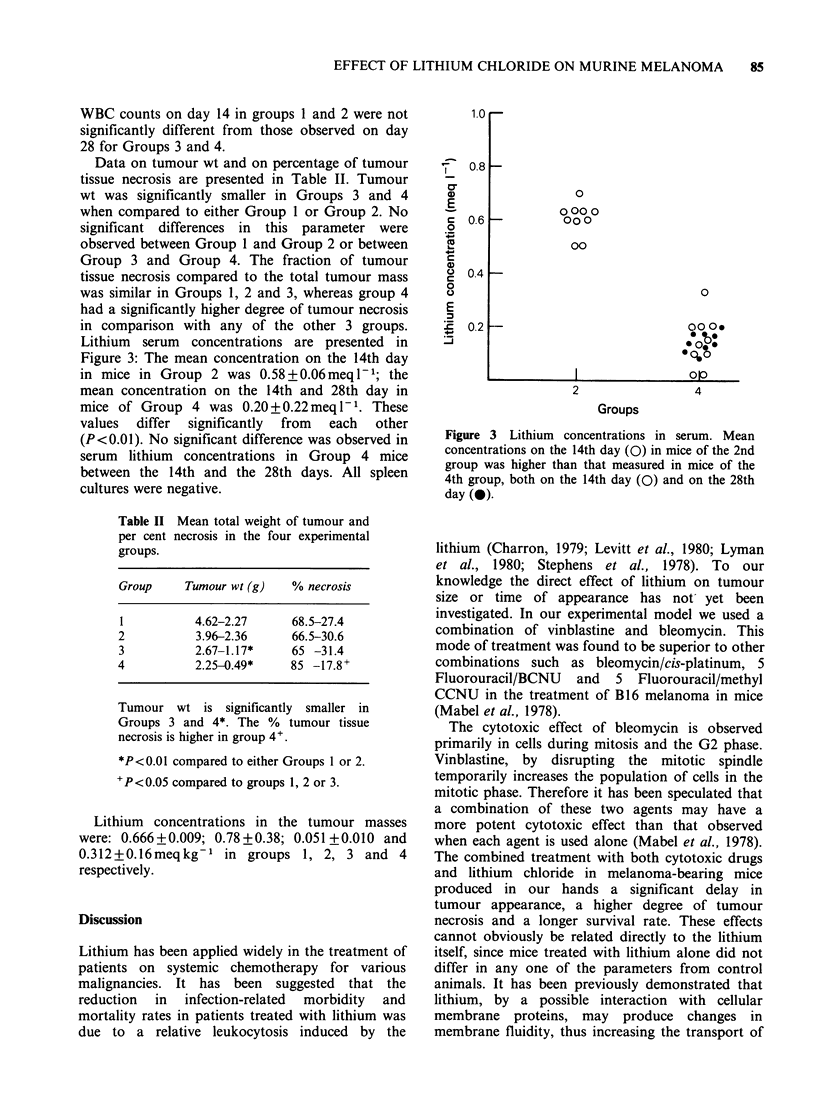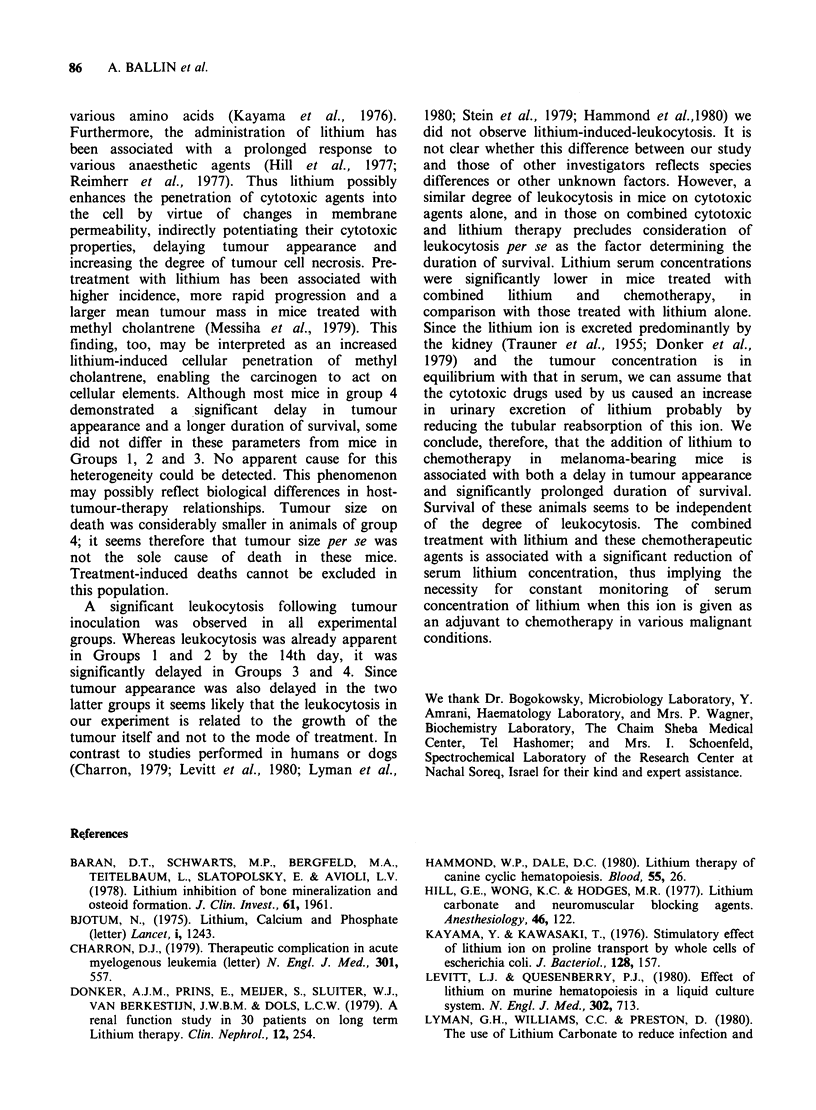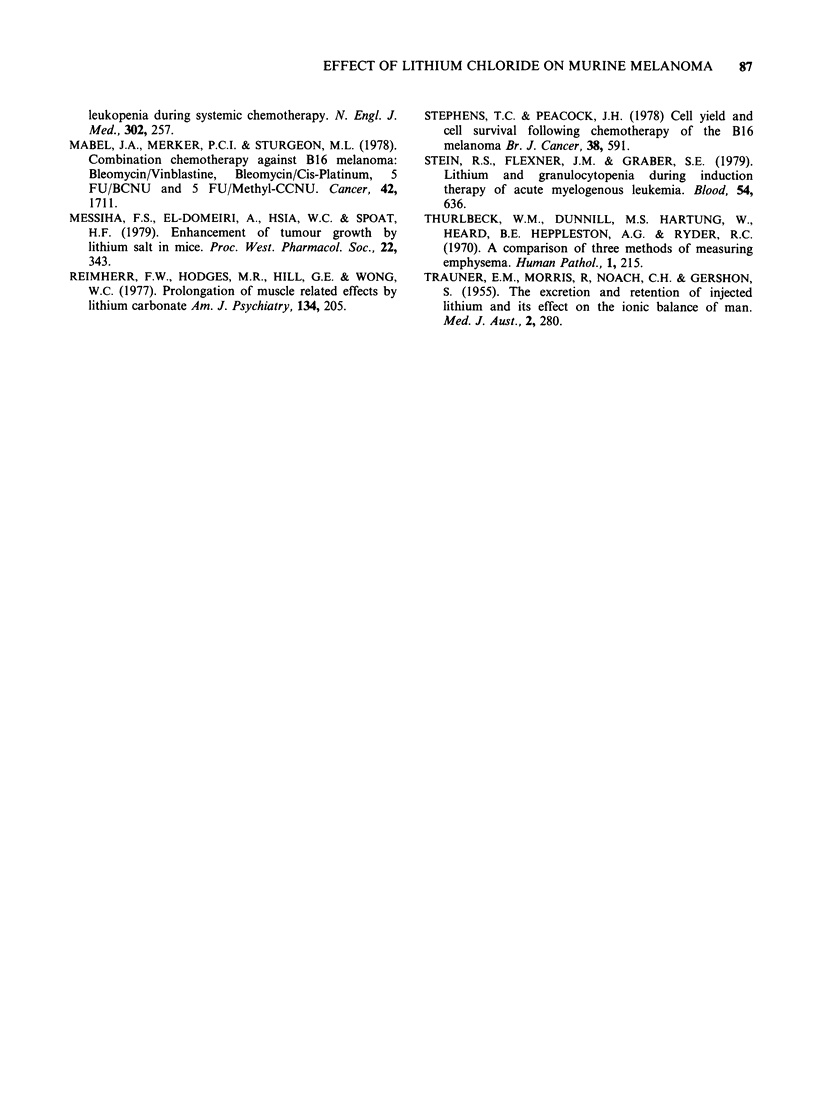# The effect of lithium chloride on tumour appearance and survival of melanoma-bearing mice.

**DOI:** 10.1038/bjc.1983.160

**Published:** 1983-07

**Authors:** A. Ballin, M. Aladjem, M. Banyash, H. Boichis, Z. Barzilay, R. Gal, I. P. Witz

## Abstract

The possible effect of lithium chloride, a compound which reduces the incidence of infection in cancer patients, was investigated on murine melanoma. C57 BL syngeneic mice were inoculated i.p. with B16 melanoma cells. The animals were divided into 4 groups, receiving daily i.p. treatment with saline--group 1, controls; lithium chloride--group 2, bleomycin and vinblastine--group 3, and lithium chloride with bleomycin and vinblastine--group 4. Animals in group 4 had a significant delay in tumour appearance, a higher degree of tumour necrosis, and a longer survival rate. In addition a significant reduction of serum lithium concentration was noted in animals of this group in comparison with animals in group 2, treated with lithium chloride alone. There was no lithium-induced leukocytosis.


*Br. J. Cancer* (1983), **48**, 83–87

### The effect of lithium chloride on tumour appearance and survival of melanoma-bearing mice

A. Ballin[1], M. Aladjem[2], M. Banyash, H. Boichis[1], Z. Barzilay[1], R. Gal & I.P. Witz

[1]Departments of Haematology and Paediatrics, The Chaim Sheba; [2]Assaf Harofeh Medical Centres and The Sackler School of Medicine and The George S. Wise Faculty of Life Sciences, Tel-Aviv University, Tel-Aviv, Israel.

**Summary** The possible effect of lithium chloride, a compound which reduces the incidence of infection in cancer patients, was investigated on murine melanoma. C57 BL syngeneic mice were inoculated i.p. with B16 melanoma cells. The animals were divided into 4 groups, receiving daily i.p. treatment with saline—group 1, controls; lithium chloride—group 2; bleomycin and vinblastine—group 3, and lithium chloride with bleomycin and vinblastine—group 4. Animals in group 4 had a significant delay in tumour appearance, a higher degree of tumour necrosis, and a longer survival rate. In addition a significant reduction of serum lithium concentration was noted in animals of this group in comparison with animals in group 2, treated with lithium chloride alone. There was no lithium-induced leukocytosis.

It has been previously reported that the administration of lithium to patients with malignancies, treated with chemotherapeutic drugs is associated with a reduced incidence of infection and infection-related death (Charron, 1979; Levitt *et al.*, 1980; Lyman *et al.*, 1980 and Stein *et al.*, 1979). This beneficial effect has been attributed to the elevated white blood counts observed in patients treated with lithium, which in turn, have been related to the stimulatory effect of this ion on pluripotent stem cells in the bone marrow (Levitt, 1980). No data on the possible effect of lithium either on tumour size or on the time of appearance of tumours have been presented to date. In view of its heterogeneous action on different tissues (Bjotum, 1975; Baran *et al.*, 1978) we investigated the leukocyte count, tumour size, time of appearance of the tumour and the survival rate in melanoma-bearing mice on chemotherapy alone and on combined treatment with chemotherapy and lithium.

### Materials and methods

The experiments were carried out in C57 BL adults female syngeneic mice, kept 10 to a cage in the animal house of the "Life Sciences Faculty", at Tel-Aviv University. Animals were divided into 4 groups, consisting of 10 mice each. Each mouse was inoculated i.p. in the right gluteal region, with $10^6$ B16 melanoma cells, obtained from Dr. J.I. Fidler, Frederick, Maryland, U.S.A. Animals in group 1 were maintained on daily i.p. injections of isotonic saline, 0.5 ml. and are henceforth referred to as controls. Animals in group 2 had daily i.p. injections of lithium chloride (Merck Co., Germany) 3 m eq $Kg^{-1}$. Animals in group 4 were treated by daily i.p. injections of lithium chloride as in group 2 in addition to the i.p. injections of Bleomycin and Vinblastine as in group 3. This was found to be the most effective mode of treatment of B16 melanoma-bearing mice (Mabel *et al.*, 1978; Stephens *et al.*, 1978). All 4 modalities of treatment were started one day after the inoculation with melanoma cells and maintained till death. Mice were examined daily and tumour presence was established by palpation.

One day before the inoculation of tumour cells and on the 14th and 28th days of treatment a blood sample was obtained from the ophthalmic venous plexus and white blood cell count and serum lithium concentration determined. In animals of groups 2 and 4, the blood sample was taken 4 h after the daily i.p. injection of lithium. Upon death, animals were weighed and the tumour was excised. Tumour excision, morphometric and histological studies were carried out by a senior pathologist (RG) who was unaware of the mode of treatment of the various animals. Tumour tissue was identified by melanoma-induced discoloration, and excised manually from the surrounding normal tissue. After weighing, tumours were fixed in 10% formol-alcohol and benzol solution and embedded

Correspondence: A. Ballin

Received 10 February 1983; Accepted 5 April 1983.

© The Macmillan Press Ltd., 1983

in paraffin. Approximately 10 sections, 5 $\mu$m thick each, were cut throughout the depth of the tumour and stained with hematoxylin and eosin. A transparent plastic grid with equidistant points, 1 mm apart, was placed over the sections. Each point was scored as either necrotic or tumour tissue. The fraction of necrotic tissue was calculated as a percentage of the cross section. Data are expressed as a mean of all studied sections (Thurlbeck *et al.,* 1970. Dunill point count). The spleens were cultured for bacterial growth using blood agar medium, MacConkey medium and Thioglycollate Broth medium.

Serum concentrations of lithium were determined by Instrumentation Laboratory, Flame Photometer 343. Lithium tissue concentrations were determined by Optical Emission Spectroscopy (I. Schoenfeld, personal communication). Statistical analysis was performed by the student's *t* test and data are expressed as means $\pm$ s.d.

### Results

There were no differences in body wt among mice in the 4 groups at the time of death. The mean and standard deviations of weights were $22.7 \pm 2.9$; $24.5 \pm 2.37$; $23.5 \pm 2.55$ and $22.8 \pm 2.15$ g for Groups 1, 2, 3 and 4 respectively.

A tumour mass was palpable in all mice in groups 1 and 2 by the tenth day, in group 3 by the 22nd day, and in group 4 by the 34th day (Figure 1). The mean time of tumour appearance in each group was $6.0 \pm 1.9$; $6.6 \pm 1.9$; $12.2 \pm 5.3$ and $23.4 \pm 9.6$ days in groups 1, 2, 3 and 4 respectively. Groups 1 and 2 did not differ significantly from each other in this respect, while significant delay in tumour appearance was observed in Group 3, when compared to groups 1 and 2 ($P < 0.05$), and group 4 differed significantly in this parameter from group 3 ($P < 0.05$) and from groups 1 and 2 ($P < 0.01$).

**Figure 1** Time interval between inoculation with B16 melanoma cells and appearance of tumour. Animals of group 1 (——) received NaCl solution; animals of group 2 (- - - -) received lithium chloride; animals of group 3 (– –) were treated with Bleomycin and Vinblastine and those of group 4 (- · · -) were treated with lithium as well as the two chemotherapeutic agents.

Duration of survival is graphically presented in Figure 2. Mean survival was $21.5 \pm 3.9$; $23.25 \pm 2.18$; $30.9 \pm 4.01$ and $38 \pm 6.0$ days for groups 1, 2, 3 and 4 respectively. No significant differences in this parameter were observed between Groups 1 and 2, however, each of these 2 groups differed significantly from either group 3 or 4 ($P < 0.01$); groups 3 and 4 also differed significantly from one another ($P < 0.05$). White blood cell counts determined on days 0, 14 and 28 of the study are presented in Table I. A significant increase in white blood count ($P < 0.01$) was observed in groups 1 and 2 on the 14th day, but there was no significant difference between the two groups. No significant change in WBC count was observed in groups 3 and 4 on the 14th day; however, by the 28th day, WBC count increased significantly in both groups.

**Figure 2** Duration of survival. Duration of survival in mice from the 4th (- · · -) group is significantly longer ($P < 0.05$) than that of mice from group 3 (– –) and much longer ($P < 0.01$) than that of mice from group 1 (——) or 2 (- - - -).

**Table I** White blood cell count ($mm^{-3}$) on days 0, 14 and 28 in the four experimental groups.

| Group \ Day | 0 | 14 | 28 |
|---|---|---|---|
| 1 | 10,800–2,500 | 22,900–11,060* | |
| 2 | 9,400–1,800 | 19,400–10,910* | |
| 3 | 9,640–1,630 | 12,200– 6,430 | 20,780– 9,180* |
| 4 | 7,900–1,520 | 9,200– 2,500 | 37,800–18,400* |

WBC Counts on day 14 in Groups 1 and 2 are not significantly different from those observed on day 28 in Groups 3 and 4.

*$P < 0.01$

WBC counts on day 14 in groups 1 and 2 were not significantly different from those observed on day 28 for Groups 3 and 4.

Data on tumour wt and on percentage of tumour tissue necrosis are presented in Table II. Tumour wt was significantly smaller in Groups 3 and 4 when compared to either Group 1 or Group 2. No significant differences in this parameter were observed between Group 1 and Group 2 or between Group 3 and Group 4. The fraction of tumour tissue necrosis compared to the total tumour mass was similar in Groups 1, 2 and 3, whereas group 4 had a significantly higher degree of tumour necrosis in comparison with any of the other 3 groups. Lithium serum concentrations are presented in Figure 3: The mean concentration on the 14th day in mice in Group 2 was $0.58 \pm 0.06 \, \text{meq} \, l^{-1}$; the mean concentration on the 14th and 28th day in mice of Group 4 was $0.20 \pm 0.22 \, \text{meq} \, l^{-1}$. These values differ significantly from each other ($P < 0.01$). No significant difference was observed in serum lithium concentrations in Group 4 mice between the 14th and the 28th days. All spleen cultures were negative.

**Table II** Mean total weight of tumour and per cent necrosis in the four experimental groups.

| *Group* | *Tumour wt (g)* | *% necrosis* |
|---|---|---|
| 1 | 4.62–2.27 | 68.5–27.4 |
| 2 | 3.96–2.36 | 66.5–30.6 |
| 3 | 2.67–1.17* | 65 –31.4 |
| 4 | 2.25–0.49* | 85 –17.8$^+$ |

Tumour wt is significantly smaller in Groups 3 and 4*. The % tumour tissue necrosis is higher in group 4$^+$.

*$P < 0.01$ compared to either Groups 1 or 2.

$^+P < 0.05$ compared to groups 1, 2 or 3.

Lithium concentrations in the tumour masses were: $0.666 \pm 0.009$; $0.78 \pm 0.38$; $0.051 \pm 0.010$ and $0.312 \pm 0.16 \, \text{meq} \, \text{kg}^{-1}$ in groups 1, 2, 3 and 4 respectively.

### Discussion

Lithium has been applied widely in the treatment of patients on systemic chemotherapy for various malignancies. It has been suggested that the reduction in infection-related morbidity and mortality rates in patients treated with lithium was due to a relative leukocytosis induced by the

**Figure 3** Lithium concentrations in serum. Mean concentrations on the 14th day (○) in mice of the 2nd group was higher than that measured in mice of the 4th group, both on the 14th day (○) and on the 28th day (●).

lithium (Charron, 1979; Levitt *et al.*, 1980; Lyman *et al.*, 1980; Stephens *et al.*, 1978). To our knowledge the direct effect of lithium on tumour size or time of appearance has not yet been investigated. In our experimental model we used a combination of vinblastine and bleomycin. This mode of treatment was found to be superior to other combinations such as bleomycin/*cis*-platinum, 5 Fluorouracil/BCNU and 5 Fluorouracil/methyl CCNU in the treatment of B16 melanoma in mice (Mabel *et al.*, 1978).

The cytotoxic effect of bleomycin is observed primarily in cells during mitosis and the G2 phase. Vinblastine, by disrupting the mitotic spindle temporarily increases the population of cells in the mitotic phase. Therefore it has been speculated that a combination of these two agents may have a more potent cytotoxic effect than that observed when each agent is used alone (Mabel *et al.*, 1978). The combined treatment with both cytotoxic drugs and lithium chloride in melanoma-bearing mice produced in our hands a significant delay in tumour appearance, a higher degree of tumour necrosis and a longer survival rate. These effects cannot obviously be related directly to the lithium itself, since mice treated with lithium alone did not differ in any one of the parameters from control animals. It has been previously demonstrated that lithium, by a possible interaction with cellular membrane proteins, may produce changes in membrane fluidity, thus increasing the transport of various amino acids (Kayama *et al.*, 1976). Furthermore, the administration of lithium has been associated with a prolonged response to various anaesthetic agents (Hill *et al.*, 1977; Reimherr *et al.*, 1977). Thus lithium possibly enhances the penetration of cytotoxic agents into the cell by virtue of changes in membrane permeability, indirectly potentiating their cytotoxic properties, delaying tumour appearance and increasing the degree of tumour cell necrosis. Pre-treatment with lithium has been associated with higher incidence, more rapid progression and a larger mean tumour mass in mice treated with methyl cholantrene (Messiha *et al.*, 1979). This finding, too, may be interpreted as an increased lithium-induced cellular penetration of methyl cholantrene, enabling the carcinogen to act on cellular elements. Although most mice in group 4 demonstrated a significant delay in tumour appearance and a longer duration of survival, some did not differ in these parameters from mice in Groups 1, 2 and 3. No apparent cause for this heterogeneity could be detected. This phenomenon may possibly reflect biological differences in host-tumour-therapy relationships. Tumour size on death was considerably smaller in animals of group 4; it seems therefore that tumour size *per se* was not the sole cause of death in these mice. Treatment-induced deaths cannot be excluded in this population.

A significant leukocytosis following tumour inoculation was observed in all experimental groups. Whereas leukocytosis was already apparent in Groups 1 and 2 by the 14th day, it was significantly delayed in Groups 3 and 4. Since tumour appearance was also delayed in the two latter groups it seems likely that the leukocytosis in our experiment is related to the growth of the tumour itself and not to the mode of treatment. In contrast to studies performed in humans or dogs (Charron, 1979; Levitt *et al.*, 1980; Lyman *et al.*, 1980; Stein *et al.*, 1979; Hammond *et al.*,1980) we did not observe lithium-induced-leukocytosis. It is not clear whether this difference between our study and those of other investigators reflects species differences or other unknown factors. However, a similar degree of leukocytosis in mice on cytotoxic agents alone, and in those on combined cytotoxic and lithium therapy precludes consideration of leukocytosis *per se* as the factor determining the duration of survival. Lithium serum concentrations were significantly lower in mice treated with combined lithium and chemotherapy, in comparison with those treated with lithium alone. Since the lithium ion is excreted predominantly by the kidney (Trauner *et al.*, 1955; Donker *et al.*, 1979) and the tumour concentration is in equilibrium with that in serum, we can assume that the cytotoxic drugs used by us caused an increase in urinary excretion of lithium probably by reducing the tubular reabsorption of this ion. We conclude, therefore, that the addition of lithium to chemotherapy in melanoma-bearing mice is associated with both a delay in tumour appearance and significantly prolonged duration of survival. Survival of these animals seems to be independent of the degree of leukocytosis. The combined treatment with lithium and these chemotherapeutic agents is associated with a significant reduction of serum lithium concentration, thus implying the necessity for constant monitoring of serum concentration of lithium when this ion is given as an adjuvant to chemotherapy in various malignant conditions.

We thank Dr. Bogokowsky, Microbiology Laboratory, Y. Amrani, Haematology Laboratory, and Mrs. P. Wagner, Biochemistry Laboratory, The Chaim Sheba Medical Center, Tel Hashomer; and Mrs. I. Schoenfeld, Spectrochemical Laboratory of the Research Center at Nachal Soreq, Israel for their kind and expert assistance.

### References

BARAN, D.T., SCHWARTS, M.P., BERGFELD, M.A., TEITELBAUM, L., SLATOPOLSKY, E. & AVIOLI, L.V. (1978). Lithium inhibition of bone mineralization and osteoid formation. *J. Clin. Invest.*, **61**, 1961.

BJOTUM, N., (1975). Lithium, Calcium and Phosphate (letter) *Lancet*, **i**, 1243.

CHARRON, D.J., (1979). Therapeutic complication in acute myelogenous leukemia (letter) *N. Engl. J. Med.*, **301**, 557.

DONKER, A.J.M., PRINS, E., MEIJER, S., SLUITER, W.J., VAN BERKESTIJN, J.W.B.M. & DOLS, L.C.W. (1979). A renal function study in 30 patients on long term Lithium therapy. *Clin. Nephrol.*, **12**, 254.

HAMMOND, W.P., DALE, D.C. (1980). Lithium therapy of canine cyclic hematopoiesis. *Blood*, **55**, 26.

HILL, G.E., WONG, K.C. & HODGES, M.R. (1977). Lithium carbonate and neuromuscular blocking agents. *Anesthesiology*, **46**, 122.

KAYAMA, Y. & KAWASAKI, T., (1976). Stimulatory effect of lithium ion on proline transport by whole cells of escherichia coli. *J. Bacteriol.*, **128**, 157.

LEVITT, L.J. & QUESENBERRY, P.J., (1980). Effect of lithium on murine hematopoiesis in a liquid culture system. *N. Engl. J. Med.*, **302**, 713.

LYMAN, G.H., WILLIAMS, C.C. & PRESTON, D. (1980). The use of Lithium Carbonate to reduce infection and leukopenia during systemic chemotherapy. *N. Engl. J. Med.*, **302**, 257.

MABEL, J.A., MERKER, P.C.I. & STURGEON, M.L. (1978). Combination chemotherapy against B16 melanoma: Bleomycin/Vinblastine, Bleomycin/Cis-Platinum, 5 FU/BCNU and 5 FU/Methyl-CCNU. *Cancer,* **42,** 1711.

MESSIHA, F.S., EL-DOMEIRI, A., HSIA, W.C. & SPOAT, H.F. (1979). Enhancement of tumour growth by lithium salt in mice. *Proc. West. Pharmacol. Soc.*, **22,** 343.

REIMHERR, F.W., HODGES, M.R., HILL, G.E. & WONG, W.C. (1977). Prolongation of muscle related effects by lithium carbonate *Am. J. Psychiatry,* **134,** 205.

STEPHENS, T.C. & PEACOCK, J.H. (1978) Cell yield and cell survival following chemotherapy of the B16 melanoma *Br. J. Cancer,* **38,** 591.

STEIN, R.S., FLEXNER, J.M. & GRABER, S.E. (1979). Lithium and granulocytopenia during induction therapy of acute myelogenous leukemia. *Blood,* **54,** 636.

THURLBECK, W.M., DUNNILL, M.S. HARTUNG, W., HEARD, B.E. HEPPLESTON, A.G. & RYDER, R.C. (1970). A comparison of three methods of measuring emphysema. *Human Pathol.,* **1,** 215.

TRAUNER, E.M., MORRIS, R, NOACH, C.H. & GERSHON, S. (1955). The excretion and retention of injected lithium and its effect on the ionic balance of man. *Med. J. Aust.*, **2,** 280.